# Relaxation of Rat Aorta by Farrerol Correlates with Potency to Reduce Intracellular Calcium of VSMCs

**DOI:** 10.3390/ijms15046641

**Published:** 2014-04-17

**Authors:** Xiaojiang Qin, Xiaomin Hou, Mingsheng Zhang, Taigang Liang, Jianmin Zhi, Lingge Han, Qingshan Li

**Affiliations:** 1 School of Pharmaceutical Science, Shanxi Medical University, No. 56, Xinjian Road, Taiyuan 030001, Shanxi, China; E-Mails: sxykdxyxy@163.com (X.Q.); ltaigang@gmail.com (T.L.); hanlg2001@126.com (L.H.); 2 School of Public Health, Shanxi Medical University, No. 56, Xinjian Road, Taiyuan 030001, Shanxi, China; 3 Department of Pharmacology, Shanxi Medical University, No. 56, Xinjian Road, Taiyuan 030001, Shanxi, China; E-Mails: houxiaominyaoli@163.com (X.H.); zmspharmacol@sina.com (M.Z.); 4 School of Physiology Science, Shanghai Jiao Tong University, No. 280, Chongqing Road, Shanghai 200240, China; E-Mail: zhjm169@gmail.com

**Keywords:** farrerol, rat aorta, VSMCs, vasorelaxation

## Abstract

Farrerol, isolated from *Rhododendron dauricum* L., has been proven to be an important multifunctional physiologically active component, but its vasoactive mechanism is not clear. The present study was performed to observe the vasoactive effects of farrerol on rat aorta and to investigate the possible underlying mechanisms. Isolated aortic rings of rat were mounted in an organ bath system and the myogenic effects stimulated by farrerol were studied. Intracellular Ca^2+^ ([Ca^2+^]_in_) was measured by molecular probe fluo-4-AM and the activities of L-type voltage-gated Ca^2+^ channels (LVGC) were studied with whole-cell patch clamp in cultured vascular smooth muscle cells (VSMCs). The results showed that farrerol significantly induced dose-dependent relaxation on aortic rings, while this vasorelaxation was not affected by N^G^-nitro-l-arginine methylester ester or endothelium denudation. In endothelium-denuded aortas, farrerol also reduced Ca^2+^-induced contraction on the basis of the stable contraction induced by KCl or phenylephrine (PE) in Ca^2+^-free solution. Moreover, after incubation with verapamil, farrerol can induce relaxation in endothelium-denuded aortas precontracted by PE, and this effect can be enhanced by ruthenium red, but not by heparin. With laser scanning confocal microscopy method, the farrerol-induced decline of [Ca^2+^]_in_ in cultured VSMCs was observed. Furthermore, we found that farrerol could suppress Ca^2+^ influx via LVGC by patch clamp technology. These findings suggested that farrerol can regulate the vascular tension and could be developed as a practicable vasorelaxation drug.

## 1. Introduction

Hypertension-associated cardiovascular events is one of the main causes of morbidity and mortality around the world [1,2]. Hypertension has become one of the most urgent public health problems [2,3], 25% of adults are suffering from this disease and this number will reach 29% by 2025 [4]. Lowering blood pressure greatly reduces the risks of developing heart failure, coronary diseases, renal damages and cerebral vascular diseases [5].

Although there are many drugs currently approved to treat hypertension, such as indapamide, metoprolol, enalapril, losartan, nifedipine [6], most of these drugs present serious adverse effects, inefficacy in term of blood pressure control, toxicity and warnings for hypertensive patients [7–10]. On the other hand, some traditional Chinese medicines (TCM), known for their good therapeutic performance and fewer side effects, are widely used for the prevention and treatment of hypertension disease [11–15]. It is well known that flavonoids can be regarded as the major active components in TCM.

In the last several decades, it has been reported that many flavonoid compounds have significant potential to prevent and treat hypertension diseases [16–21]. Flavanone as a subclass of flavonoids consist of a large group of naturally existing polyphenolic compounds widely distributed in plants [22,23]. Previous studies have demonstrated that some flavonones, such as hesperetin and naringenin present relaxation effects on the rat aorta [24–26]. Farrerol, a typical natural flavanone, has been isolated from *Rhododendron dauricum* L., and has been extensively used to alleviate symptoms associated with bronchial asthma in China [27]. Accumulating evidence suggested that farrerol possesses many biological properties, including antibechic, antibacterial, anti-inflammatory effects, and an inhibititory effect on VSMCs proliferation [28]. Furthermore, our previous study showed that farrerol presents a strong anti-atherosclerosis activity in VSMCs and exhibited a significant cytoprotective activity against hydrogen peroxide (H_2_O_2_)-induced injury in human umbilical vein endothelial cells, indicating its potential to treat and prevent cardiovascular diseases [29]. In order to shed light on its therapeutic potential for the treatment of hypertension diseases, we explored the effects of farrerol on the rat aorta, as well as the possible mechanisms involved.

## 2. Results and Discussion

### 2.1. Results

#### 2.1.1. Dose-Dependent Relaxation Effect of Farrerol

**Figure 1.**
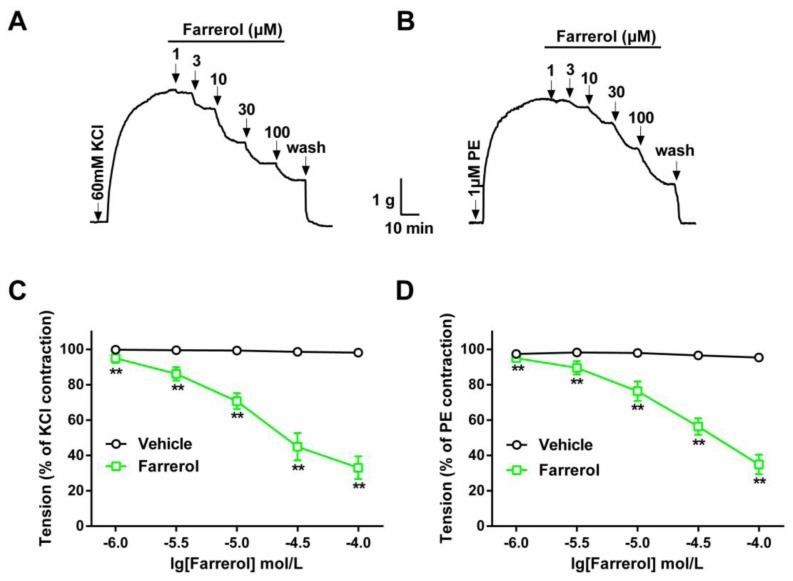
Dose-dependent relaxant effects of farrerol on isolated rat aortic rings. Typical original traces of the effects of farrerol on KCl- (**A**) or PE-induced (**B**) contractions. The vertical arrows indicate drug addition. Concentration-dependent effects of farrerol (1–100 μM ) on KCl- (**C**) or PE-precontracted (**D**) rat aortic rings. The Student’s *t*-test was used in the statistical analysis. Data are expressed as means ± SD (*n* = 6–8). ** *p* < 0.01, *vs*. vehicle.

As shown in Figure 1, contractions induced by 60 mM KCl or 1 μM PE on rat isolated aortic rings were 3.53 ± 0.32 and 3.27 ± 0.29 g, respectively. Farrerol induced relaxation on aortic rings with KCl- or PE-induced contraction in a dose-dependent manner. At a concentration of 100 μM, farrerol induced the maximal relaxations of 66.97% for KCl and 65.13% for PE, respectively. The experiment indicated that there was a slight or no difference in the potency or sensitivity of the farrerol-induced relaxation effect in rings contracted by KCl or PE. The respective EC_50_ values were 14.02 μM for KCl and 35.94 μM for PE.

**Figure 2.**
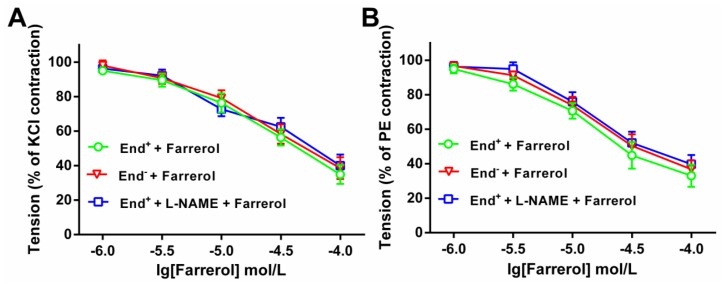
Farrerol-induced vasorelaxation in endothelium-intact (End^+^) or -denuded (End^−^) rat aorta rings precontracted with KCl (**A**) or phenylephrine (PE) (**B**). When the precontraction was sustained, L-NAME (0.1 mM) was added to the bath. When the contraction was steady again in the presence of the L-NAME, the concentration-relaxation curve of farrerol was reconstructed. Relaxations were expressed as percentages of the precontracton induced by 60 mM KCl or 1 μM PE, respectively. The statistical comparisons were made using one-way ANOVA followed by Newman-Keuls test. Data are expressed as means ± SD (*n* = 7–8). * *p* < 0.05, *vs*. End^+^ + Farrerol.

Moreover, Figure 2 indicates that the relaxation effect of farrerol did not differ in endothelium-intact and -denuded aortas. Preincubation with L-NAME also did not affect the relaxation. Thus the following experiments were carried out in endothelium-denuded aortas and VSMCs.

#### 2.1.2. Effect of Farrerol on Extracellular Ca^2+^-Induced Contraction

The endothelium-denuded aorta rings contraction induced by KCl (60 mM) was mainly due to the depolarization of VSMCs and the influx of extracellular Ca^2+^ through voltage-gated Ca^2+^ channels [30,31]. In high-K^+^, Ca^2+^-free Krebs’ solution, cumulative addition of CaCl_2_ (1–100 μM) induced a gradually increased tension of aortic rings. Pretreatment with farrerol at 14.02 μM noticeably depressed the maximal contraction to 53.90% ± 6.67%.

**Figure 3.**
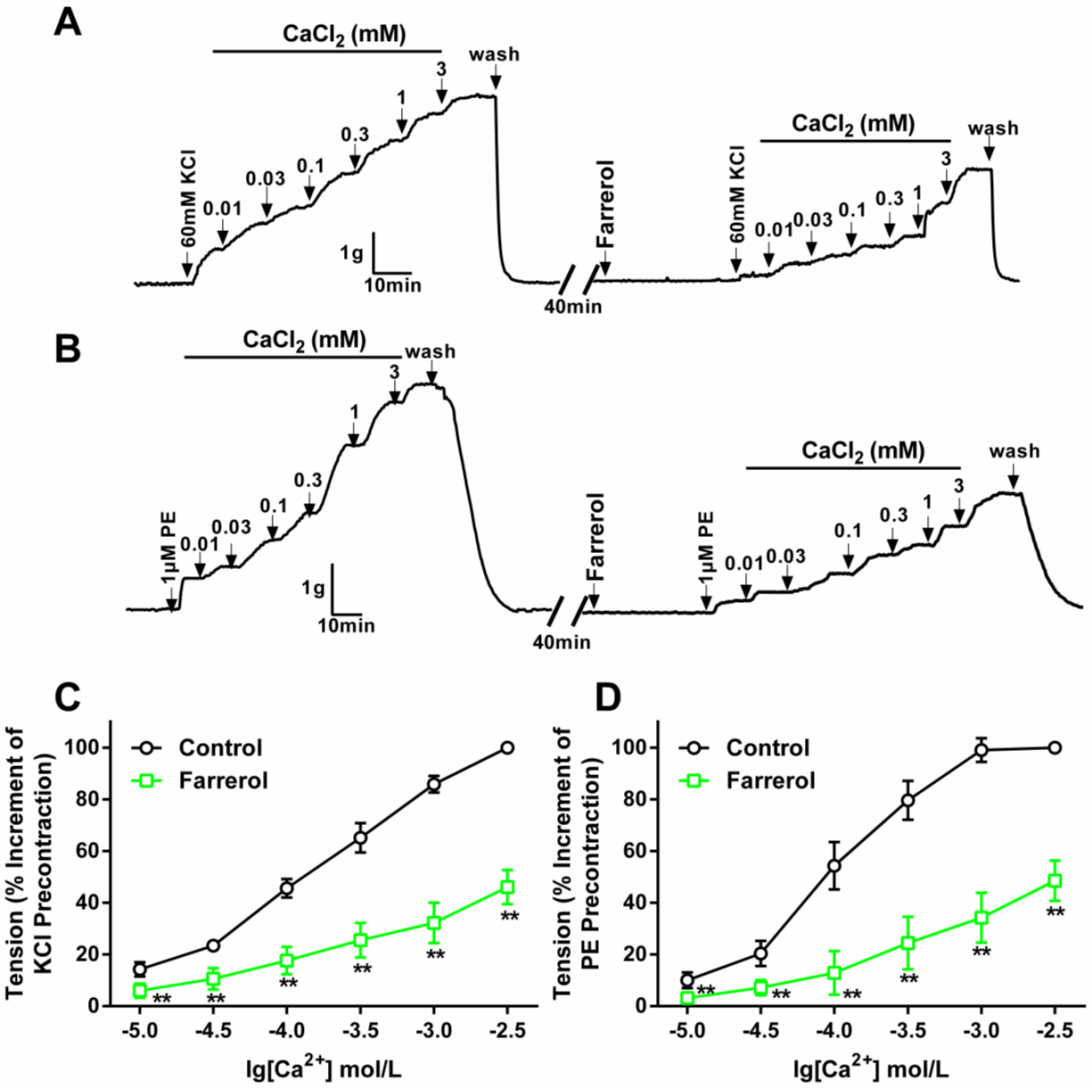
Effect of farrerol on extracellular Ca^2+^-induced contractions on rat ndothelium-denuded aortic rings. (**A**,**B**) Original tension tracings of the effects of giving prior farrerol or not on KCl- (**A**) or PE-induced (**B**) contractions. Drug addition is indicated by the vertical arrows. Contractions were presented as gram (g); and (**C**,**D**) The maximal contraction induced by CaCl_2_ was taken as 100%. Results are expressed as means ± SD (*n* = 6–8). ** *p* < 0.01, *vs*. control. The statistical analysis used the Student’s *t*-test.

The contraction induced by PE was mainly caused by the influx of extracellular Ca^2+^ through the receptor-operative Ca^2+^ channel (ROCC) [32,33]. The effect of farrerol on the ROCC was studied in Ca^2+^-free Krebs’ solution; PE (1 μM) pre-stimulated endothelium-denuded aortic rings were used. Farrerol (35.94 μM) pre-incubation significantly inhibited the contraction induced by extracellular CaCl_2_ (0.01–3 mM), and the maximum contraction was decreased to 51.43% ± 7.87%, as shown in Figure 3.

#### 2.1.3. Relationship between Vasoactive Effects of Farrerol and [Ca^2+^]_in_ Release

**Figure 4.**
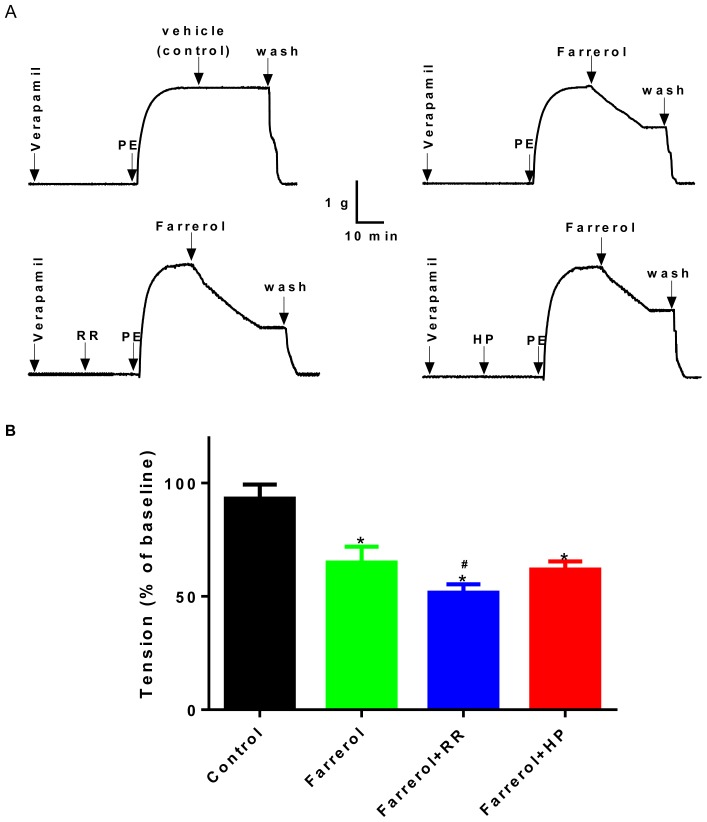
Effects of farrerol at 35.94 μM in rat aortic ring contracted with PE (1 μM) after verapamil (1 μM) pretreatment in the absence (control) or the presence of ruthenium red (RR) (10 μM) or heparin (HP) (50 mg/L). (**A**) Original tension tracings; (**B**) Data are expressed as means ± SD. (*n* = 7–10). * *p* < 0.05, *vs*. control; ^#^ *p* < 0.05, *vs.* farrerol. The statistical analysis used the Anova with Dunnet’s *post hoc* test.

In this experiment, endothelium-denuded rings were pretreated for 30 min with 1 μM Verapamil, an L-type Ca^2+^ channel inhibitor, and subsequently contracted by PE. Except in the control (vehicle) group, farrerol (35.94 μM) was added in another three experimental groups, and then the curves of relaxation were clearly shown. In addition, this effect of farrerol was enhanced by 10 μM ruthenium red (RR), a ryanodine receptor inhibitor (*p* < 0.01 compared to farrerol only), but not by 50 mg/L heparin (HP), an IP_3_ receptor inhibitor (Figure 4). The result demonstrated that the effect of farrerol on vasorelaxation was related to the ryanodine receptor.

#### 2.1.4. Effects of Farrerol on [Ca^2+^]_in_ in Rat Aortic Vascular Smooth Muscle Cells (VSMCs)

**Figure 5.**
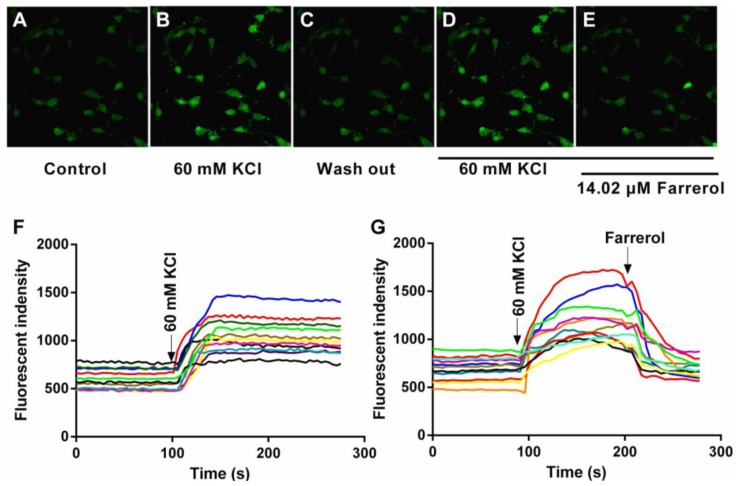
Effect of farrerol on 60 mM KCl-induced elevation of [Ca^2+^]_in_ in rat aortic VSMCs. [Ca^2+^]_in_ profiles of VSMCs following addition of farrerol assayed by Fluo 4-AM using a laser scanning confocal microscope at room temperature. The fluorescence intensities change was plotted to represent the change in [Ca^2+^]_in_ levels. (**A**–**E**) Original fluorescence images (×400). (**A**) before KCl was added (control); (**B**) 60 mM KCl was added; (**C**) wash out KCl with physiological salt solution; (**D**) 60 mM KCl was added; (**E**) 14.02 μM farrerol was added; and (**F**,**G**) representative original tracing of relative fluorescence intensity of [Ca^2+^]_in_ in each treatment (arrow indicates the time point when 60 mM KCl or 14.02 μM farrerol was added).

Laser scanning confocal microscopy method was employed to investigate [Ca^2+^]_in_ changes in cultured VSMCs. Our preliminary experiments showed that 60 mM KCl was optimal for measuring [Ca^2+^]_in_ Typical [Ca^2+^]_in_ profiles were shown in Figure 5. The fluorescence intensity increased by 100.6% after addition of KCl which means that KCl could significantly increase [Ca^2+^]_in_. Farrerol (14.02 μM) was then applied to the cells and we observed a decrease of 72.9% in the intracellular fluorescence intensity. The VSMCs were then washed with 60 mM KCl in order to remove farrerol while keeping the cells constantly exposed to 60 mM KCl. KCl induced again an increase of [Ca^2+^]_in_, gradually restoring the calcium levels to the basal level measured before the application of farrerol. The above results indicate that farrerol could decrease [Ca^2+^]_in_ in cultured VSMCs.

#### 2.1.5. Effects of Farrerol on [Ca^2+^]_ex_ Influx Current through L-Type Voltage-Gated Ca^2+^ Channels (LVGC) in Rat Aortic VSMCs

**Figure 6.**
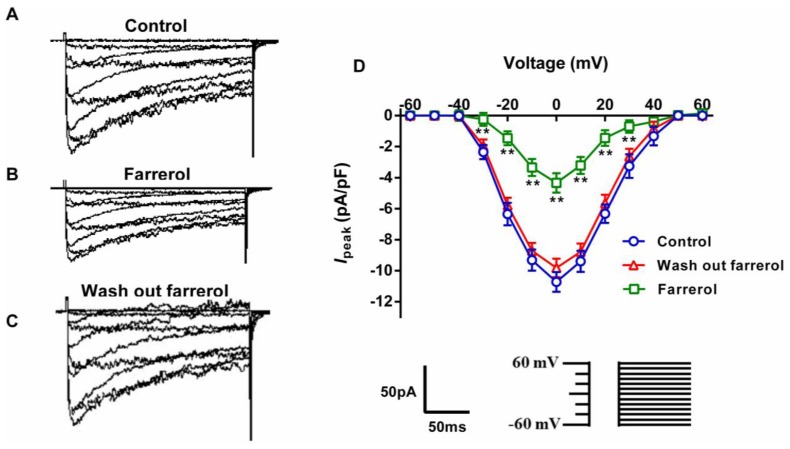
Effects of farrerol on the *I*_Ba,L_ current-voltage (I–V) curves. (**A**–**C**) original tracings; (**D**) I–V relationships of the inward currents evoked by a series of depolarizing pulses (from −60 to +60 mV in 10 mV increments, duration 200 ms) in the absence (control, wash out farrerol) or the presence of farrerol (14.02 μM), respectively. Data are expressed as mean ± SD from VSMCs isolated from 8 rats. ** *p* < 0.01, *vs*. control.

The inhibitory potency of farrerol on Ca^2+^ influx current was comparable to the intracellular fluorescence intensity decrement and its inhibitory potency on the contraction. We hypothesized that the inhibitory effect of farrerol on the aorta contraction and on the elevation of free intracellular Ca^2+^ in aortic VSMCs may be because of its inhibitory action at the level of LVGC. Thus, the effects of farrerol on [Ca^2+^]_ex_ influx currents were measured using whole-cell patch clamp technique. In order to obtain currents of a bigger magnitude, Ba^2+^ was used as the charge carrier. Currents appeared at a voltage of about −40 mV, and peaked at around 0 mV. The maximal current density was −10.72 ± 0.45 pA/pF (*n* = 8). At a test potential of 0 mV, 14.02 μM farrerol reduced the Ca^2+^ current by 59.50% (Figure 6).

### 2.2. Discussion

Farrerol, a flavonone, isolated from *Rhododendron dauricum* L. (known as man-shan-hong in Chinese), has been extensively used for the treatment of bronchitis and asthma in China. The present study revealed that farrerol has vasorelaxation properties. This is the first study of the myogenic and electric effects of farrerol on rat aorta.

We hypothesized that farrerol acts through a reduction in Ca^2+^ availability, which was confirmed by three different experiments in the present study. Myogenic experiments showed that farrerol mainly inhibited the contraction component induced by [Ca^2+^]_ex_ influx. The calcium assays demonstrated that farrerol inhibited the [Ca^2+^]_in_ elevation induced by depolarization. Moreover, the patch clamp experiments indicated that farrerol reduced [Ca^2+^]_ex_ influx currents through LVGC, which is also consistent with previous studies showing that inhibiting the entry of [Ca^2+^]_ex_ is a contributing mechanism to the vasorelaxation induced by farrerol. Ca^2+^ plays essential roles in regulating the vascular tone [34]. A reduction in Ca^2+^, can lead to relaxation in VSMCs. Such a reduction is caused by a prevention of Ca^2+^ entry from the extracellular fluid or a reduction of Ca^2+^ release from the intracellular Ca^2+^ store [35–37].

Farrerol, at 1–100 μM, could markedly induce relaxation of KCl- and PE-induced contraction of aortic rings in a dose-dependent manner, with EC_50_ values of 14.02 μM for KCl and 35.94 μM for PE. At the same time, our results showed that farrerol-induced vasorelaxation was not affected by endothelium denudation and L-NAME (0.1 mM), suggesting that neither rat aorta endothelium nor NO production is necessary to the rat aorta vasorelaxation. Here, we found that farrerol (1–100 μM) evoked relaxation in aortic rings precontracted by KCl and PE regardless of the presence or absence of endothelium. This indicated that farrerol acted directly on VSMCs to induce relaxation, and not by endothelium-derived vasodilator factors. Based on our results, the experiments were carried out in endothelium-denuded aortas and VSMCs.

It is well known that a high concentration of extracellular KCl leads to the depolarization of the cell membrane, which induces the increase in transmembrane Ca^2+^ influx at the opening of LVGC and subsequently leads to vascular smooth muscles contraction [38]. Farrerol (100 μM), showed a maximal relaxation of 66.97%, suggesting that inhibiting LVGC may mediate farrerol-induced relaxation. Furthermore, farrerol (14.02 μM) reduced the contraction of aortic rings when KCl produced a steady contraction followed by gradually increasing Ca^2+^ input in Ca^2+^-free Krebs’ solution. The results also indicated that farrerol blockade of LVGC decrease in the influx of [Ca^2+^]_ex_ may be another critical mechanism involved in the relaxation. The fact that farrerol can reduce the increase in [Ca^2+^]_in_ elicited by KCl suggests that farrerol may inhibit voltage-dependent Ca^2+^ channels activity, resulting in decreased Ca^2+^ entry and [Ca^2+^]_in_. The measurements of [Ca^2+^]_in_ confirmed this observation.

Previous studies showed that PE, an α-adrenoreceptor agonist, causes aortic contraction by Ca^2+^ influx through ROC and by release of Ca^2+^ from the sarcoplasmic reticulum [39–42]. In this manuscript, farrerol (100 μM) induced a maximal relaxation of 65.13%, implying that farrerol may block ROC and inhibit Ca^2+^ release from sarcoplasmic reticulum stores to decrease [Ca^2+^]_in_ and relax the aorta. At the same time, farrerol (35.94 μM) was observed to inhibit contraction when PE produced a steady contraction followed by gradual Ca^2+^ input in a Ca^2+^-free Krebs’ solution, which indicated that farrerol blockade of ROC to decrease the influx of [Ca^2+^]_ex_ may be a critical mechanism in relaxing the aorta.

We found that farrerol (35.94 μM ) induced relaxation both in the endothelium-denuded and -intact aortas pretreated for 30 min with 1 μM verapamil and subsequently contracted by PE. This effect of farrerol, enhanced by 10^−5^ M ruthenium red, but not by 50 mg/L heparin, suggested that the contraction action of farrerol on aorta may be mediated by blocking ryanodine receptors to release Ca^2+^ from the sarcoplasmic reticulum.

Taken together, our results suggest that farrerol relaxes contracted rat aorta through the involvement of LVGC as well as ROC. Furthermore, the relaxation effect of farrerol may be mediated by blocking ryanodine receptors to release Ca^2+^ from the sarcoplasmic reticulum. These results suggest that farrerol may be a possible therapeutic agent for the treatment of hypertension diseases.

## 3. Experimental Section

### 3.1. Chemicals and Drugs

**Figure 7.**
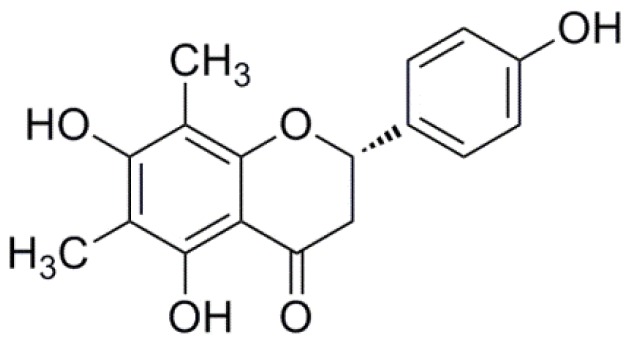
Chemical structure of farrerol.

Ethylene glycol-bis (2-aminoethylether)-*N*,*N*,*N*′,*N*′-tetraacetic acid (EGTA), acetylcholine chloride, and phenylephrine (PE) were purchased from Sigma (St. Louis, MO, USA). 4-(2-Hydroxyethyl) piperazine-1-ethanesulfonic acid (HEPES) was purchased from AMRESCO Company (Solon, OH, USA). Verapamil, ruthenium red (RR), and heparin (HP) were from Sigma Company. Potassium chloride (KCl), sodium chloride (NaCl), magnesium chloride (MgCl_2_), glucose, calcium chloride (CaCl_2_) and all the other reagents were of analytical purity. Farrerol (Figure 7) was prepared according to the reported method [29]. The purity of the compound was higher than 98%. They were dissolved in dimethylsulfoxide (DMSO) and then diluted in distilled water in order to yield a final concentration of DMSO inferior to 0.5% (*v*/*v*), which has been shown to be devoid of any observable effects on the vascular muscle tone and rat aorta VSMCs.

### 3.2. Animals

Male Sprague Dawley rats (weight, 250 ± 20 g) were fed a diet containing 11% fat, 60% vegetable starch, and 29% protein, supplied by the Animal Center of Shanxi Medical University. Rats were housed in cages, had free access to food and water, and were maintained in a 12 h light/dark cycle. All protocols and procedures of our experiments described were approved by the Animal Care and Use Committee of the Shanxi Medical University (Taiyuan, China), and all efforts were made to minimize the number of animals used and their suffering, in accordance with the ethical guidelines for animal research at Shanxi Medical University.

### 3.3. Aorta Preparations and General Protocols

Male Sprague Dawley rats were anesthetized by intraperitoneal administration of sodium pentobarbitol (40 mg/kg) and then euthanized by exsanguination. The aorta was isolated and immediately transferred into a chilled (4 °C) Krebs’ solution, composed of (mM): NaCl, 118.0; KCl, 4.7; MgSO_4_·7H_2_O, 1.2; KH_2_PO_4_, 1.2; CaCl_2_, 2.5; NaHCO_3_, 25.0; and Glucose, 11.0, bubbled with 95% O_2_ + 5% CO_2_ (pH 7.4). The aortas were cleaned of adherent connective tissues and cut into rings of 4 mm length. The rings were suspended on two wire hooks in water-jacketed tissue baths containing 5 mL of the Krebs’ solution kept at 37 °C and gassed with 95% O_2_ + 5% CO_2_. The upper hook was connected to a force transducer and changes in isometric force were recorded using Chart 5.4 (PowerLab, AD Instruments Co, Ltd., Dunedin, New Zealand) and saved on a computer hard disk. Passive tension was adjusted to 2 g and all subsequent measurements representing the force were generated above this baseline [43]. A 2 h equilibration period was applied before any experimental intervention, during which the bath was flushed with fresh Krebs’ solution every 15 min [44]. After equilibration, the rings were activated three times with Krebs’ solution containing 60 mM KCl for 10 min. We assumed integrity of the endothelium when 10^−6^ M acetylcholine induced greater than 70% relaxation of the aortic rings with KCl-induced (60 mM) contraction in a reproducible manner, when the variations of the measured values were inferior to 10% between successive contractions or relaxation. We then assessed the effects of the drugs. In some experiments, the endothelium was mechanically removed by gently rubbing the artery lumen with a steel wire [45]. The rings were allowed to rest for at least 40 min after each contraction. For the relaxation experiments, the rings were pretreated with 1 μM PE or 60 mM KCl, in order to induce about 80% of the maximal contraction [46].

#### 3.3.1. Effect of Farrerol on KCl- and PE-Induced Contraction of Rat Aortic Rings

We evaluated the effects of farrerol on the relaxation of rat aortic rings. PE and KCl were used to induce steady contraction in intact rings. The farrerol was added cumulatively within the following range: 1–100 μM. The farrerol-induced dose-dependent relaxation of aortic rings was calculated as a percentage of PE- or KCl-induced contraction.

To explore the possible mechanisms, the effect of farrerol in endothelium-denuded rings was compared with that in endothelium-intact rings to study involvement of the endothelium in farrerol-induced rat aortic rings relaxation. At the same time, the effects of inhibitors on farrerol-induced rat aorta relaxation were studied using 0.1 mM L-NAME [47,48], a NO synthesis inhibitor. When the contraction induced by 60 mM KCl or 1 μM PE reached a sustained plateau, L-NAME was added to the chamber. Ten minutes later, farrerol was stepwise added to the chamber if the arterial tone in the presence of L-NAME was stable. Relaxation was expressed as a percentage of the precontraction induced by 60 mM KCl or 1 μM PE, respectively.

#### 3.3.2. Effect of Farrerol on the Endothelium-Denuded Aortic Rings Contraction Induced by [Ca^2+^]_in_ Release and [Ca^2+^]_ex_ Influx

In the first step of experiments, we investigated the role of Ca^2+^ influx in farrerol-induced relaxation. Endothelium-denuded aortic rings were washed 2–3 times with Ca^2+^-free Krebs’ solution (containing 1 μM EGTA). After the tone of the rings returned to baseline, PE (1 μM) or KCl (60 mM) was applied to produce steady contraction. Ca^2+^ was then added cumulatively in order to obtain a concentration-response curve (0.01–3 mM). The EC_50_ of farrerol was incubated 30 min before addition of PE or KCl. The bath concentration of farrerol was kept constant throughout the following experiments.

In the second step of experiments, the aim was to expound whether the relaxation of farrerol was related to the inhibition of [Ca^2+^]_in_ release. Endothelium-denuded aortic rings were exposed to 10^−6^ M verapamil [49] for 30 min before the application of 1 μM PE to induce a steady contraction; subsequently the EC_50_ of farrerol was added to evoke a relaxation. 10^−5^ M ruthenium red [50,51] or 50 mg/L heparin [52] was added 15 min before the application of 1 μM PE in a separate experimental group.

### 3.4. VSMCs Preparation

**Figure 8.**
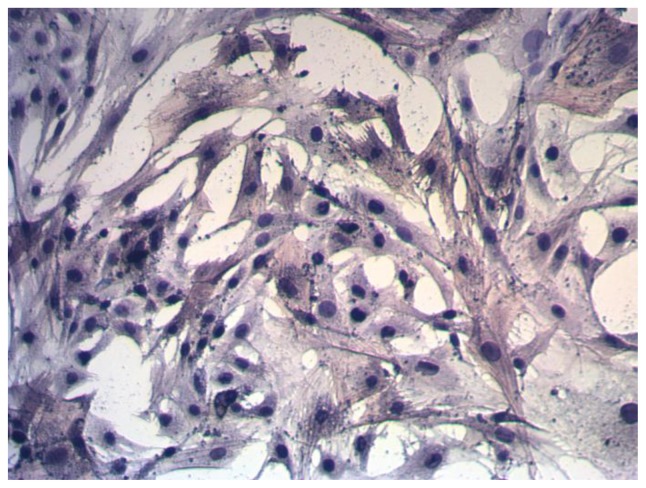
The pure identification of rat aortic VSMCs (×100).

After the aortas were isolated and the endothelial cells (ECs) were removed, the smooth muscle layer was stripped and chopped into small fragments (approximately 1 mm^3^) in 0.5 mL of FBS. The fragments, together with the FBS, were transferred to a 25 cm^2^ flask and maintained upside down in an incubator at 37 °C for 4 h. The flask was turned over gently and incubated for four to seven days after addition of 2 mL of DMEM. VSMCs migrated out of the explants four to seven days later, and passage was performed 10–14 days after isolation. The cultures were maintained in DMEM containing 10% FBS, penicillin 100 U/mL, and streptomycin 100 mg/L. The medium was refreshed every 48 h, and cells were passaged when they reached 60% confluence. When the cells displayed a typical “hill and valley” growth appearance and after characterization using immunostaining against α-smooth muscle actin, passages 4–7 was used for experiments (Figure 8).

#### 3.4.1. [Ca^2+^]_in_ Measurements

VSMCs were plated on dishes and loaded with 2 μM Fluo-4-AM for 30 min at 37 °C in a physiological salt solution (145 mM NaCl, 3 mM KCl, 2 mM CaCl_2_, 1 mM MgCl_2_, 10 mM glucose, 10 mM HEPES and 1% BSA; pH 7.4). VSMCs were then washed with physiological salt solution for 15 min at 37 °C in order to remove the extracellular excess of dye. Fluorescence intensity (fluorescence intensity after 60 mM KCl, 60 mM KCl + farrerol and 60 mM KCl in order of time precedence) were recorded. [Ca^2+^]_in_ measurements were carried out using an OLYMPUS FV1000 (Tokyo, Japan) confocal microscope at room temperature. Fluo-4-AM was excited at 494 nm, and the emission was recorded at 516 nm; the measured fluorescence was subtracted from the background and autofluorescence. The fluorescent intensity was used to represent the changes in intracellular Ca^2+^ levels.

#### 3.4.2. Whole-Cell Patch Clamp Recordings

Whole cell L-type Ca^2+^ currents were recorded from single aorta VSMCs. In order to obtain the bigger magnitude currents, Ba^2+^ was used as the charge carrier. Cells were allowed to settle for 40 min and then were gently washed with a bath solution in order to remove the debris of cell and tissue from the chamber. The bath solution contained 135 mM NaCl, 5 mM KCl, 2 mM MgCl_2_, 5 mM HEPES, and 10 mM Glucose, and the pH was set at 7.4. The average access resistance was 4–8 MΩ, and the cell capacitance was 8–12 pF. Patch pipettes were pulled from borosilicate capillaries using a PP-830 vertical puller (Narishige, Tokyo, Japan). Patch pipettes were filled with a solution containing 130 mM CsCl, 2 mM MgCl_2_, 5 mM Mg-ATP (Adenosine triphosphate), 5 mM HEPES, 10 mM Glucose and 10 mM EGTA at pH 7.2. CsCl was included in the pipettes solution to eliminate K^+^ currents. The voltage protocol was designed according to the characteristics of *I*_Ba_ in rat aortic VSMCs. *I*_Ba_ was measured at holding potentials of −60 mV and subjected to step depolarizations of 200 ms to +60 mV in 10 mV increments every 1 s. Voltage-activated calcium currents were recorded under a flowing bath solution containing 80 mM BaCl_2_, 30 mM TEA (Tetraethylammonium chloride), 2 mM MgCl_2_, 5 mM HEPES and 10 mM Glucose at pH 7.4. Data were collected after stabilization of the current amplitude, usually 1–2 min after the whole-cell configuration had been obtained. The input resistance of the cells must exceed 1 GΩ.

### 3.5. Statistical Analysis

All data are presented as means ± SD. The letter “*n*” represents the number of rats. The EC_50_ was defined as the concentration of farrerol that induced 50% of the maximum relaxation from the contraction elicited by PE (1 μM) or KCl (60 mM) and was calculated from the concentration–response curve resulting from a nonlinear regression (curve fit) performed using GraphPad Prism (Version 6.0, GraphPad Software Inc., La Jolla, CA, USA). Statistical analysis was done using the Student’s *t*-test and one-way analysis of variance (ANOVA). In all cases, statistical significance was defined when a *p* value of <0.05 was obtained. Laser confocal data were analyzed with the FV10-ASW 3.0 software (Tokyo, Japan). Patch clamp data were analyzed by the softwares IGOR Pro 6.1 (Wavemetrics, Lake Oswego, OR, USA) and PULSEFIT (HEKA Electronik, Bremen, Germany).

## 4. Conclusions

In conclusion, farrerol could markedly induce relaxation of rat aortic rings. The involvement of LVGC as well as ROC contributes to the relaxant effects of farrerol in rat aorta. Furthermore, this activity correlated well with its potency to reduce [Ca^2+^]_in_ in VSMCs. These data provide strong evidence that farrerol inhibits both entry [Ca^2+^]_ex_ and release of intracellular stored Ca^2+^ in VSMCs, and suggest that farrerol has potential effects on the regulation of the hypertension and could be developed as a practicable vasorelaxation drug.
